# OA02 Paediatric chronic pain and Autism Spectrum Disorder: is there a link?

**DOI:** 10.1093/rap/rkac066.002

**Published:** 2022-09-28

**Authors:** Gabriella Jones, Vinay Shivamurthy

**Affiliations:** Rheumatology and Chronic Pain Service, Evelina London Children's Hospital, London, United Kingdom; Rheumatology and Chronic Pain Service, Evelina London Children's Hospital, London, United Kingdom

## Abstract

**Introduction/Background:**

To review the literature on chronic pain and autism spectrum disorder (ASD). A key aim is to explore the prevalence of chronic pain in children with ASD and the impact that ASD could have on the experience of chronic pain.

**Description/Method:**

After reviewing the literature, a thematic analysis was completed in order to identify key patterns. The thematic analysis identified four key topics: Prevalence of chronic pain in ASD; Impact of comorbid ASD and chronic pain; Psychological flexibility; Sensory sensitivities.

**Discussion/Results:**

The literature demonstrates that the prevalence of chronic pain is higher in children with ASD than the general population; studies show the prevalence of pain to be almost doubled in children with ASD (ASD = 15.6%; without ASD = 8.2%). Chronic pain can also have a more significant impact on functioning in children who exhibit ASD traits, shown through higher pain interference, higher depression levels and lower health related quality of life. This greater functional impairment means interventions are particularly important, however, challenges are highlighted by the literature; ASD traits may affect the efficacy of talking therapies and treatment guidelines are not specific, such as those for comorbid ASD and anxiety not being intended for use with physical health conditions.

An important protective factor identified in comorbid chronic pain and ASD is psychological flexibility; acting on long-term values rather than current thoughts and feelings. However, this is typically low in ASD thus highlighting a role for Acceptance and Commitment Therapy (ACT), which aims to increase psychological flexibility. This is supported in chronic pain research where ACT leads to improved functioning, particularly in those with higher ASD traits.

A discrepancy exists in chronic pain literature as there is a higher prevalence of children with clinically significant ASD traits than with confirmed ASD diagnosis. It appears that medically unexplained pain can interfere with ASD diagnosis, with it often being the first presenting symptom in undiagnosed ASD, and oversensitivity to pain has been associated with delayed ASD diagnosis. Sensory and perceptual abnormalities are commonly seen in ASD, with research indicating an underlying link between hypersensitivity and pain. However, current pain sensitivity findings can often not be generalised to children with comorbid chronic pain and ASD; studies tend to exclude those with such physical health conditions and investigate only acute pain sensitivity in ASD.

**Key learning points/Conclusion:**

There is a link between chronic pain and ASD evident in the literature as children with ASD are at a higher risk of developing chronic pain than the general population, particularly if they also experience abnormal or hypersensitivities.

Chronic pain is likely to have a greater impact on children with ASD, including higher depression levels and lower quality of life.

Due to the risk for greater functional impairment, it is particularly important to manage chronic pain in children with ASD, however common ASD traits and a lack of specific guidelines pose challenges in doing so.

Due to a lack of existing research, further research is required to gain understanding of the link between chronic pain and ASD, including exploring sensory abnormalities in chronic pain and the management of comorbid chronic pain and ASD.

It is important raise awareness of this association amongst clinicians, particularly due to the prevalence of undiagnosed ASD, the impact on intervention, and the risk for more severe functional impairment in paediatric chronic pain.

